# Dietary butyrate ameliorates metabolic health associated with selective proliferation of gut *Lachnospiraceae bacterium 28-4*

**DOI:** 10.1172/jci.insight.166655

**Published:** 2023-02-22

**Authors:** Zhuang Li, Enchen Zhou, Cong Liu, Hope Wicks, Sena Yildiz, Farhana Razack, Zhixiong Ying, Sander Kooijman, Debby P.Y. Koonen, Marieke Heijink, Sarantos Kostidis, Martin Giera, Ingrid M.J.G. Sanders, Ed J. Kuijper, Wiep Klaas Smits, Ko Willems van Dijk, Patrick C.N. Rensen, Yanan Wang

**Affiliations:** 1 Department of Medicine, Division of Endocrinology, and; 2 Einthoven Laboratory for Experimental Vascular Medicine, Leiden University Medical Center, Leiden, Netherlands.; 3 Microbiome Medicine Center, Department of Laboratory Medicine, Zhujiang Hospital, Southern Medical University, Guangzhou, China.; 4 Department of Pediatrics, University Medical Center Groningen, Groningen, Netherlands.; 5 Center for Proteomics and Metabolomics,; 6 Department of Medical Microbiology,; 7 Center for Microbiome Analyses and Therapeutics, and; 8 Department of Human Genetics, Leiden University Medical Center, Netherlands.; 9 Med-X Institute, Center for Immunological and Metabolic Diseases, and Department of Endocrinology, the First Affiliated Hospital of Xi’an JiaoTong University, Xi’an Jiaotong University, Xi’an, China.

**Keywords:** Endocrinology, Microbiology, Obesity

## Abstract

Short-chain fatty acids, including butyrate, have multiple metabolic benefits in individuals who are lean but not in individuals with metabolic syndrome, with the underlying mechanisms still being unclear. We aimed to investigate the role of gut microbiota in the induction of metabolic benefits of dietary butyrate. We performed antibiotic-induced microbiota depletion of the gut and fecal microbiota transplantation (FMT) in APOE*3-Leiden.CETP mice, a well-established translational model for developing human-like metabolic syndrome, and revealed that dietary butyrate reduced appetite and ameliorated high-fat diet–induced (HFD-induced) weight gain dependent on the presence of gut microbiota. FMT from butyrate-treated lean donor mice, but not butyrate-treated obese donor mice, into gut microbiota–depleted recipient mice reduced food intake, attenuated HFD-induced weight gain, and improved insulin resistance. 16S rRNA and metagenomic sequencing on cecal bacterial DNA of recipient mice implied that these effects were accompanied by the selective proliferation of *Lachnospiraceae bacterium 28-4* in the gut as induced by butyrate. Collectively, our findings reveal a crucial role of gut microbiota in the beneficial metabolic effects of dietary butyrate as strongly associated with the abundance of *Lachnospiraceae bacterium 28-4*.

## Introduction

Obesity is becoming a global health concern. Although lifestyle interventions, including calorie restriction (1) and pharmacotherapy, have been shown to be effective in inducing weight loss (2), cessation of intervention generally leads to weight regain. Therefore, intervention strategies aimed at attaining sustained weight loss are still required.

Dietary fiber intake is associated with lower BW, lower incidence of cardiometabolic diseases, and lower mortality from type 2 diabetes (T2D) and coronary heart disease (3). One of the main mechanisms attributed to the cardiometabolic benefits of dietary fiber is the production of short-chain fatty acids (SCFAs), including acetate, propionate, and butyrate, by microbial fermentation (4). Particularly, butyrate was shown to prevent diet-induced obesity (DIO) (5), improve glucose homeostasis, and alleviate insulin resistance in mice (6). Moreover, we previously demonstrated that dietary butyrate prevents high-fat diet–induced (HFD-induced) weight gain in mice mainly via reducing food intake, in addition to modestly increasing energy expenditure by activating brown adipose tissue (BAT) (7). A recent clinical study, however, showed that oral butyrate improves glucose metabolism only in lean individuals but not in subjects with metabolic syndrome (8). Further investigation of the precise molecular targets of butyrate in various metabolic contexts is, therefore, necessary to provide insight into the differential response of individuals with and without obesity upon butyrate intervention, which may lead to the identification of personalized therapeutic strategies to combat obesity and associated cardiometabolic diseases in the clinic.

A groundbreaking human intervention study showed that adding fiber to an isoenergetic diet beneficially alters the gut microbiota by promoting SCFA-producing bacterial strains, which alleviates T2D as evident from the improvement of HbA1c (9). In addition, a recent human study demonstrated that the overall gut microbiota shifts in parallel with glycemic status, suggesting that the variation of gut microbiota is strongly associated with insulin resistance of the host (10). Collectively, these findings indicate that gut microbiota plays a crucial role in maintaining cardiometabolic health.

Here, we addressed the role of gut microbiota in the induction of metabolic benefits of dietary butyrate by using antibiotic-induced microbiota depletion (AIMD) of the gut and fecal microbiota transplantation (FMT) in APOE*3-Leiden.CETP (E3L.CETP) mice, a well-established translational model for developing human-like diet-induced cardiometabolic diseases (11). We reveal that the beneficial metabolic effects of dietary butyrate are crucially dependent on gut microbiota. In particular, FMT from butyrate-treated lean donor mice, but not from butyrate-treated obese donor mice, induces enrichment of *Lachnospiraceae bacterium 28-4* in recipient mice, positively correlating with their cardiometabolic health.

## Results

### Dietary butyrate reduces food intake and attenuates HFD-induced weight gain dependent on gut microbiota.

**Figure 1:**
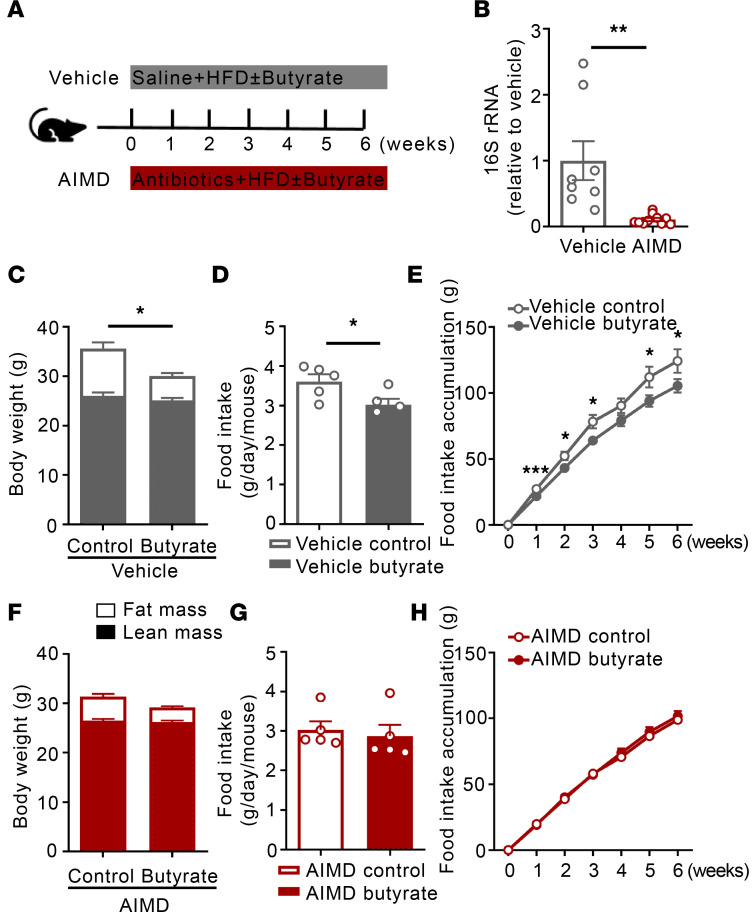
Dietary butyrate reduces food intake and attenuates HFD-induced weight gain dependent on gut microbiota. (**A**) Mice underwent AIMD or received saline (vehicle) for 6 weeks while being fed an HFD without or with 5% (weight by weight [w/w], i.e., on average 0.12 g per day per mouse) sodium butyrate. (**B**) At the end of the treatment, fresh feces were collected and bacterial DNA was quantified by 16S rRNA gene amplification by PCR (*n* = 8–9). (**C** and **F**) Body composition was measured by MRI (*n* = 8). (**D** and **G**) The average food intake per day throughout the whole intervention period was calculated (*n* = 5). (**E** and **H**) The cumulative food intake was calculated (*n* = 5). Data are shown as means ± SEM; statistical significance between 2 groups was determined with 2-tailed Student’s unpaired *t* test. For data represented in the line graphs showing the changes over time for a continuous variable, statistical significance between 2 groups at each time point was determined using 2-tailed Student’s unpaired *t* test. *P < 0.05, **P < 0.01; AIMD vs. vehicle in **B** or Butyrate vs. Control in **C**–**H**.

Our previous work suggested that butyrate alters gut microbiota composition, which may contribute to the beneficial effects of butyrate on metabolic health (7). Therefore, we first explored the role of gut microbiota in the metabolic benefits of dietary butyrate. To this end, male E3L.CETP mice that underwent AIMD or received saline (vehicle) were simultaneously fed an HFD without or with sodium butyrate for 6 weeks (Figure 1A). Compared with vehicle, AIMD largely reduced 16S rRNA expression (–95%, *P* < 0.01) in fresh fecal samples collected after the intervention (Figure 1B), verifying depletion of the bacterial gut microbiota. In the vehicle group, butyrate administration attenuated HFD-induced fat mass gain (–49%, *P* < 0.05) without affecting lean mass (Figure 1C), as explained by reduced daily (–15%, *P* < 0.05) and cumulative (–13%, *P* < 0.05) food intake (Figure 1, D and E). AIMD abolished the effects of butyrate on fat mass gain (Figure 1F) and food intake (Figure 1, G and H), indicating that the induction of the metabolic benefits by dietary butyrate is strictly dependent on the presence of gut microbiota.

Additionally, we examined the role of gut microbiota in the effects of dietary butyrate on energy expenditure, lipid metabolism, and the activity of BAT, a key regulator in energy hemostasis (12). In the vehicle group, dietary butyrate decreased the respiratory exchange ratio during the night period (–5%, *P* < 0.05; Supplemental Figure 1A; supplemental material available online with this article; https://doi.org/10.1172/jci.insight.166655DS1), as a result of the increased fat oxidation (+14%, *P* < 0.05; Supplemental Figure 1B) at the expense of carbohydrate oxidation (–28%, *P* < 0.01; Supplemental Figure 1C). In addition, dietary butyrate also accelerated the clearance of glycerol tri[^3^H]oleate-labeled ([^3^H]TO-labeled) triglyceride-rich lipoprotein-like (TRL-like) particles from the circulation (*P* < 0.05; Supplemental Figure 1D) and increased the uptake of [^3^H]TO-derived radioactivity by BAT (+110%, *P* < 0.05; Supplemental Figure 1E). Consistent with our previous findings (7), butyrate activated BAT and enhanced BAT thermogenic capacity, as evidenced by reducing intracellular lipid content (Supplemental Figure 1, F and I) as well as increasing protein expression of both uncoupling protein-1 (UCP-1) (Supplemental Figure 1, G and I) and tyrosine hydroxylase (TH), a marker of sympathetic nerve activity (Supplemental Figure 1, H and I). In contrast, in the AIMD group, butyrate neither affected the respiratory exchange ratio (Supplemental Figure 1J), fat oxidation, and carbohydrate oxidation rate (Supplemental Figure 1, K and L) nor influenced the [^3^H]TO clearance from the circulation and the tissue uptake of [^3^H]TO-derived radioactivity (Supplemental Figure 1, M and N). AIMD also abolished the effects of butyrate on BAT activation (Supplemental Figure 1, O–R).

### FMT transplantation from butyrate-treated lean donor mice attenuates HFD-induced weight gain and improves insulin resistance in recipient mice.

**Figure 2:**
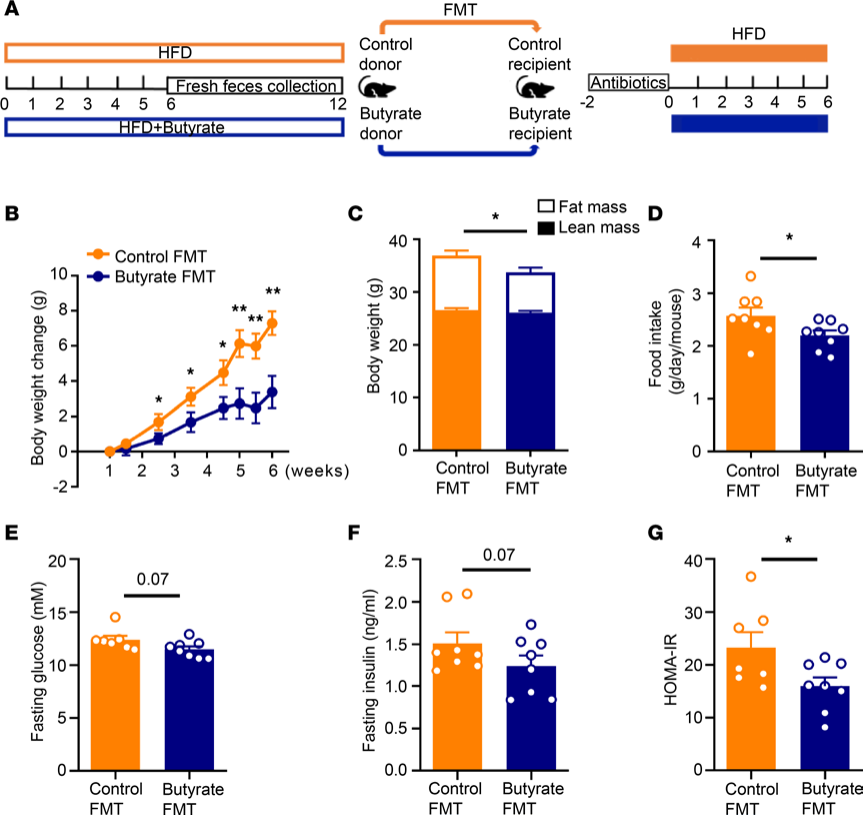
FMT from butyrate-treated lean donor mice attenuates HFD-induced weight gain and improves insulin resistance in recipient mice. (**A**) Mice were fed an HFD without or with 5% (w/w) sodium butyrate prevention for 6 weeks. After this, fresh feces were collected weekly and used for FMT to gut microbiota-depleted recipient mice that were fed an HFD for 6 weeks. (**B**) BW was measured weekly and the BW change was calculated (*n* = 8). (**C**) At the end of the experiment, body composition was measured by MRI (*n* = 8). (**D**) The average food intake per day throughout the intervention period was calculated (*n* = 8). (**E**) Fasting glucose (*n* = 7–8) and (**F**) insulin (*n* = 8) plasma levels were measured. (**G**) They were then used for calculation of HOMA-IR (*n* = 7–8). Data are shown as means ± SEM; statistical significance between 2 groups was determined with 2-tailed Student’s unpaired *t* test; For data represented in the line graphs showing the changes over time for a continuous variable, statistical significance between 2 groups at each time point was determined using 2-tailed Student’s unpaired *t* test. *P < 0.05, **P < 0.01; Butyrate vs. Control.

To further elucidate the causal impact of gut microbiota on the metabolic benefits of dietary butyrate, donor mice were fed an HFD without (Control) or with butyrate (Butyrate) for 12 weeks (prevention strategy), and from week 6 onward, fresh fecal bacteria were isolated from donors and transplanted to gut microbiota–depleted mice upon HFD feeding for 6 weeks (Figure 2A). Compared with mice receiving FMT from control donors, FMT from butyrate-treated donors caused a persistent decrease in BW gain (–54% at 6 weeks, *P* < 0.01; Figure 2B), accompanied by a decrease in fat mass (–26%, *P* < 0.05; Figure 2C), as well as a reduction in daily food intake (–15%, *P* < 0.05; Figure 2D). In addition, FMT from butyrate-treated donors tended to decrease fasting plasma levels of glucose (–11%, *P* = 0.07; Figure 2E) and insulin (–25%, *P* = 0.07; Figure 2F) and markedly reduced homeostatic model assessment of insulin resistance (HOMA-IR) (–32%, *P* < 0.05; Figure 2G) in recipient mice.

Compared with FMT from control donors, FMT from butyrate-treated donors decreased the respiratory exchange ratio in recipient mice (–3%, *P* < 0.05; Supplemental Figure 2A) accompanied by an unchanged fat oxidation rate (Supplemental Figure 2B) and decreased carbohydrate oxidation rate (–28%, *P* < 0.05; Supplemental Figure 2C). The clearance of [^3^H]TO from the circulation (Supplemental Figure 2D) and uptake of [^3^H]TO-derived radioactivity by various organs including BAT were not altered (Supplemental Figure 2E). In line with this, BAT activity was comparable between both groups, as no differences were observed for intracellular lipid content (Supplemental Figure 2, F and I), UCP-1 expression (Supplemental Figure 2, G and I), and TH expression (Supplemental Figure 2, H and I) within BAT. Collectively, these data reveal that butyrate indirectly, i.e., through modulation of the gut microbiota, causes satiety and attenuates HFD-induced weight gain and insulin resistance.

### FMT from butyrate-treated lean donor mice selectively enriches Lachnospiraceae bacterium 28-4 in recipient mice.

To unravel the specific effects of butyrate on the composition of the gut microbiota in relation to its metabolic effects on the host, 16S rRNA-Seq as well as metagenomic sequencing were performed on cecal bacterial genomic DNA of all mice receiving FMT from control or butyrate-treated donors.

**Figure 3:**
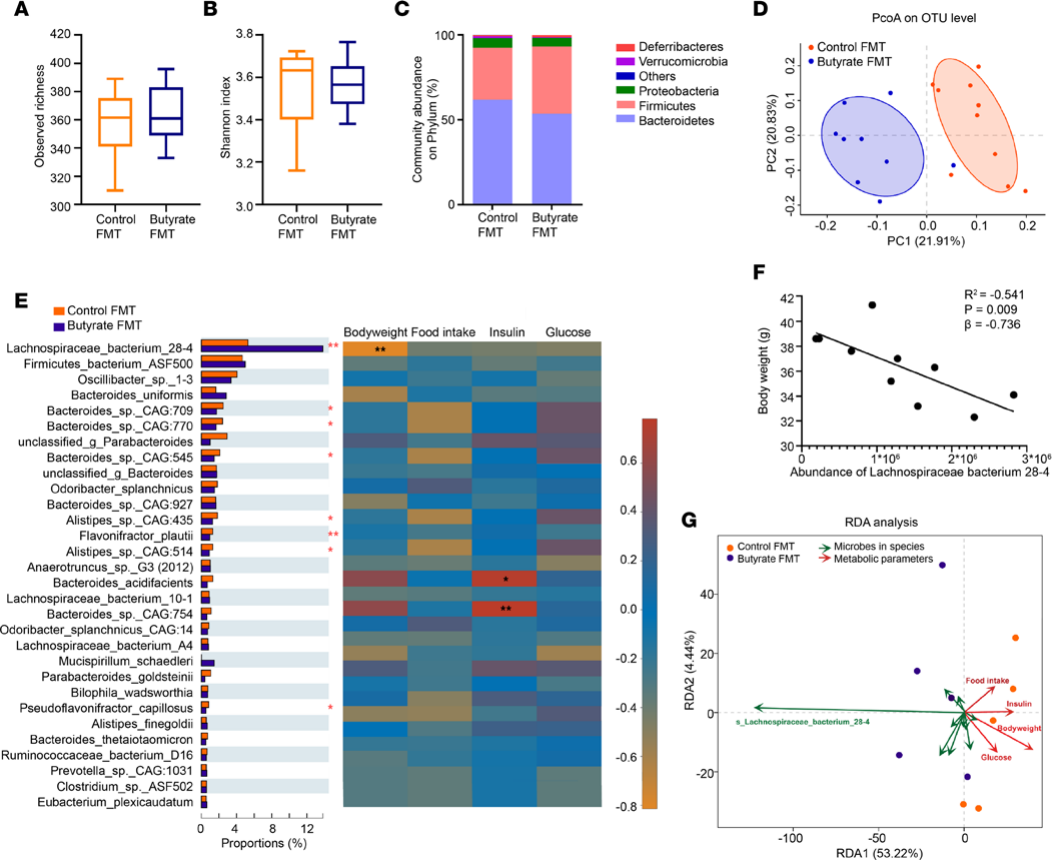
FMT from butyrate-treated lean donor mice selectively enriches *Lachnospiraceae bacterium 28-4* in recipient mice. At the end of the FMT study, the recipients’ bacterial genomic DNA was isolated from the cecum content and sequenced. By 16S rRNA gene analysis, (**A**) the number of observed species (*n* = 9–10), (**B**) Shannon diversity (*n* = 9–10), and (**C**) the community abundance of gut microbiota on phylum level (*n* = 9–10) were assessed. (**D**) The PCoA plot of unweighted UniFrac distances on OTU levels was then calculated (*n* = 9–10). (**E**) By analyzing the metagenomic sequencing data, the abundance of gut microbiota (top 30 based on relative abundance) on species level was compared using Wilcoxon’s rank-sum test and its correlation with metabolic outcomes (BW, food intake, glucose, and insulin) were presented in Spearman’s correlation heatmap (*n* = 5). (**F**) Correlation of abundance of *Lachnospiraceae bacterium 28-4* with BW was analyzed (*n* = 5). (**G**) Correlations of abundance of *Lachnospiraceae bacterium 28-4* with metabolic outcomes (BW, food intake, glucose, and insulin) were analyzed using RDA (*n* = 5). Data are shown as box plot with whiskers at min/max in **A** and **B**; statistical significance between 2 groups was determined with 2-tailed Student’s unpaired *t* test in **A** and **B** and Wilcoxon’s rank-sum test in **E**. *P < 0.05, **P < 0.01; Butyrate vs. Control.

16S rRNA-Seq analysis revealed changes in the gut microbial ecology in recipient mice. The observed richness of the operational taxonomic unit (OTU) (Figure 3A) and Shannon index (α-diversity, Figure 3B) were not different between recipient groups. In favorable contrast, FMT from butyrate-treated donors induced an apparent difference in composition of gut microbiota in recipient mice, as presented by an increase in abundance of *Firmicutes* (+28%, *P* < 0.05) at the expense of predominantly *Bacteroidetes* (–10%; Figure 3C and Supplemental Table 1), and induced different clustering in principal coordinates analysis (PCoA) plot of unweighted unique fraction metric (UniFrac) distances on the OTU level (β-diversity, Figure 3D).

Next, metagenomic analysis revealed more distinctive variations in the gut microbiota at the species level. We identified 6,840 species in total, spanning 1,851 genera and 104 phyla (Supplemental Table 2). Among those, 859 species were significantly regulated by FMT from butyrate-treated donors compared with control donors (Supplemental Table 3). In particular, among the top 30 species based on relative abundance, FMT from butyrate-treated donors markedly increased the relative abundance of *Lachnospiraceae bacterium 28-4* (+2.9-fold, *P* < 0.01), while it decreased the relative abundance of *Bacteroides sp*. *CAG:709*, *Bacteroides sp*. *CAG:770*, *Bacteroides sp*. *CAG:545*, *Alistipes sp*. *CAG:435*, *Flavonifractor plautii*, *Alistipes sp*. *CAG:514*, and *Pseudoflavonifractor capillosus* (Figure 3E) in recipient mice. Enrichment with *Lachnospiraceae bacterium 28-4* was also shown by further analysis using linear discriminant analysis of effect size (LefSe) (Supplemental Figure 3A). The comparable abundance of *Lachnospiraceae bacterium 28-4* in donor and recipient mice in control or butyrate groups indicates a successful transplantation of *Lachnospiraceae bacterium 28-4* from donor to recipient mice (Supplemental Figure 3B).

To further investigate whether butyrate could directly stimulate the proliferation of *Lachnospiraceae bacterium 28-4*, we anaerobically cultured the cecal bacterial mixture in vitro without or with 4 mM exogenous butyrate for 7 days and assessed the abundance of *Lachnospiraceae bacterium 28-4* by real-time PCR. We observed comparable Ct values for the abundance of *Lachnospiraceae bacterium 28-4* regardless of whether butyrate was added or not (Supplemental Figure 3C), suggesting that exogenous butyrate had no direct effect on the proliferation of *Lachnospiraceae bacterium 28-4* in vitro.

### The abundance of Lachnospiraceae bacterium 28-4 in the gut negatively correlates with host BW.

To elucidate whether the alteration of gut microbiota by FMT from butyrate-treated donors correlates to the metabolic health of the host, we analyzed the predicted functional contributions of the richest strains (top 30 based on relative abundance) using the Kyoto Encyclopedia of Genes and Genomes (KEGG) database (Pathway level 2; Supplemental Figure 4 and Supplemental Table 4). Correlation heatmap analysis showed the involvement of *Lachnospiraceae bacterium 28-4*, *Eubacterium plexicaudatum*, *Lachnospiraceae bacterium COE1*, and *Pseudoflavonifractor capillosus* in various aspects of host metabolic health in mice receiving FMT from control and butyrate-treated donors. Particularly, the abundance of *Lachnospiraceae bacterium 28-4* was associated with carbohydrate metabolism (F1), energy metabolism (F8), and lipid metabolism (F13; Supplemental Figure 4 and Supplemental Table 4). Consistent with this predictive analysis, functional correlation analysis using measured metabolic parameters, including BW, food intake, glucose level, and insulin level of FMT-recipient mice revealed that the abundance of *Lachnospiraceae bacterium 28-4* negatively correlated with host BW (*R*^2^ = –0.541, *P* < 0.01; Figure 3, E and F). Furthermore, redundancy analysis (RDA) supported a negative correlation between *Lachnospiraceae bacterium 28-4* and metabolic health parameters including BW, food intake, fasting plasma glucose level, and insulin level (Figure 3G). Collectively, these data provide evidence for the involvement of *Lachnospiraceae bacterium 28-4* in the metabolic benefits induced by dietary butyrate.

**Figure 4:**
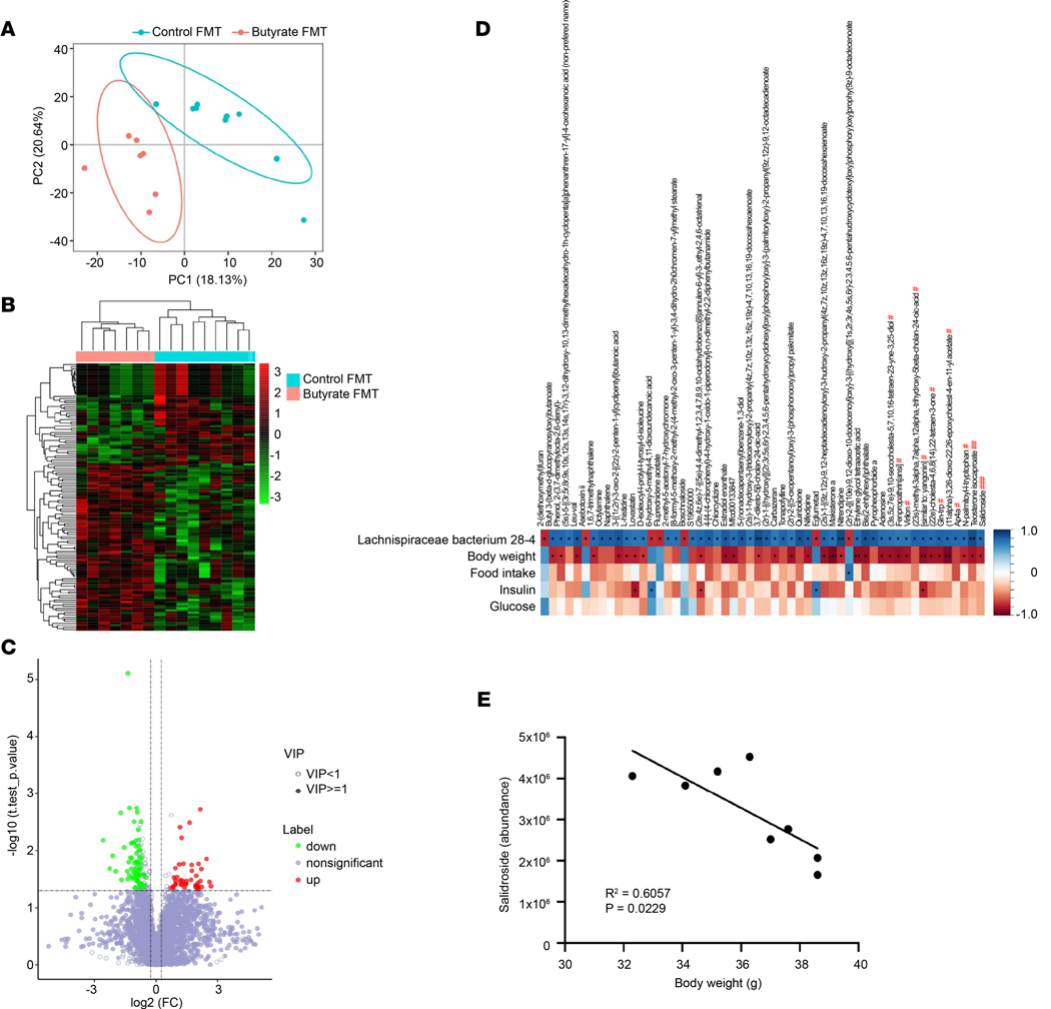
FMT from butyrate-treated lean donor mice alters the gut metabolites in recipient mice. At the end of the FMT study, the recipients’ cecal content was collected for untargeted metabolomics analysis. The differences in the composition and abundance of metabolites between mice receiving butyrate FMT and control FMT are presented by (**A**) PCoA, (**B**) hierarchical cluster analysis, and (**C**) volcano plots. (**D** and **E**) The correlation of abundance of metabolites with the abundance of *Lachnospiraceae bacterium 28-4* and metabolic outcomes (including BW, food intake, glucose, and insulin) were presented in Spearman’s correlation heatmap and analyzed. *P < 0.05, **P < 0.01, ***P < 0.001; Butyrate vs. Control. The statistical significance in the abundance of metabolites between mice receiving butyrate FMT and control FMT was determined with 2-tailed Student’s unpaired *t* test. ^#^*P* < 0.05, ^##^*P* < 0.01, ^###^*P* < 0.001. PCoA, principal coordinates analysis; VIP, variable importance in projection.

Next, we performed untargeted metabolomics analysis of cecal content samples from mice receiving FMT to characterize the differential cecal metabolites between mice receiving FMT from butyrate-treated donors and control-treated donors. The metabolomic signatures were different between groups as evidenced by the separated plots in the PCoA (Figure 4A), the hierarchy of clusters in the heatmap analysis (Figure 4B), and the variance in metabolite abundance in the volcano plots (Figure 4C). We observed that several metabolites, including fenpropathrin; virilon; (23s)-methyl-3α,7α,12α-trihydroxy-5β-cholan-24-oic acid; (22e)-cholesta-4,6,8(14),22-tetraen-3-1; Gln-Trp; (11α)-3,26-dioxo-22,26-epoxycholest-4-en-11-yl acetate; Ap4a; *N*-palmitoyl-l-tryptophan; testosterone isocaproate; and salidroside, were positively associated with the abundance of *Lachnospiraceae bacterium 28-4* (Figure 4D). For instance, the abundance of salidroside was significantly correlated with BW (Figure 4E), indicating that salidroside, which has been shown to improve glucose and insulin metabolism (13), may have a potential role in mediating the beneficial effects of *Lachnospiraceae bacterium 28-4* on metabolic health.

### Butyrate treatment does not induce weight loss, ameliorate metabolic health, or promote Lachnospiraceae bacterium 28-4 in DIO mice.

**Figure 5:**
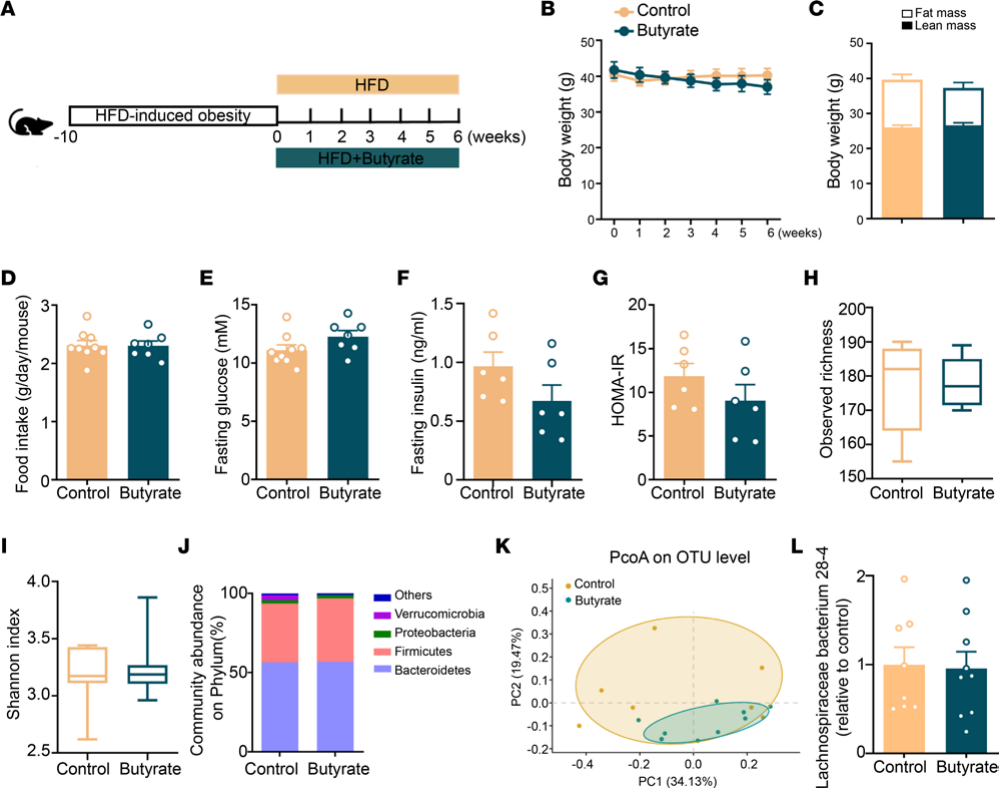
Butyrate treatment does not induce weight loss, ameliorate metabolic health, or promote *Lachnospiraceae bacterium 28-4* in DIO mice. (**A**) Mice were rendered DIO by being fed an HFD for 10 weeks and subsequently fed an HFD without or with 5% (w/w) butyrate for another 6 weeks. (**B**) BW was measured weekly (*n* = 7–9) and (**C**) body composition was measured at the end of the treatment period by MRI (*n* = 7–9). (**D**) The average food intake per day was calculated (*n* = 7–9). (**E**) Fasting plasma glucose (*n* = 7–9) and (**F**) insulin (*n* = 6) were measured and (**G**) used to calculate HOMA-IR (*n* = 6). Cecum bacterial DNA was collected for 16S rRNA-Seq and (**H**) the observed richness of taxonomy (*n* = 7–9) and (**I**) Shannon diversity (*n* = 7–9) of gut microbiota were calculated. (**J**) The composition of abundant bacteria on phylum (*n* = 7–9) and (**K**) PCoA plot of unweighted UniFrac distances on OTU level (*n* = 7–9) were calculated. (**L**) The abundance of *Lachnospiraceae bacterium 28-4* was quantified by real-time PCR (*n* = 8–9). Data are shown as means ± SEM for **B**–**G** and **L** or box plot with whiskers at min/max for **H** and **I**. Statistical significance between 2 groups was determined with 2-tailed Student’s unpaired *t* test. For data represented in the line graphs showing the changes over time for a continuous variable, statistical significance between 2 groups at each time point was determined using 2-tailed Student’s unpaired *t* test.

To investigate the therapeutic effects of dietary butyrate on the treatment of DIO, mice were first rendered obese by HFD feeding and were subsequently fed the same HFD without or with butyrate supplementation for another 6 weeks (treatment strategy; Figure 5A). In this setting, butyrate did not cause any reduction in BW (Figure 5B), fat mass (Figure 5C), food intake (Figure 5D), fasting plasma glucose (Figure 5E), fasting plasma insulin (Figure 5F), or HOMA-IR (Figure 5G). Likewise, dietary butyrate failed to alter the gut microbiota in DIO mice with respect to observed OTU abundance (Figure 5H), Shannon index (Figure 5I), and community abundance on phylum (Figure 5J), resulting in overlapping clustering between control and butyrate-treated mice in unweighted UniFrac PCoA (Figure 5K). Of note, in DIO mice, butyrate also did not promote the proliferation of *Lachnospiraceae bacterium 28-4* (Figure 5L). Taken together, these data show that butyrate does not improve metabolic health in the context of obesity, probably related to the absence of effects on gut microbiota.

### FMT from butyrate-treated obese donor mice does not attenuate weight gain, ameliorate metabolic health, or enrich Lachnospiraceae bacterium 28-4 in recipient mice.

**Figure 6:**
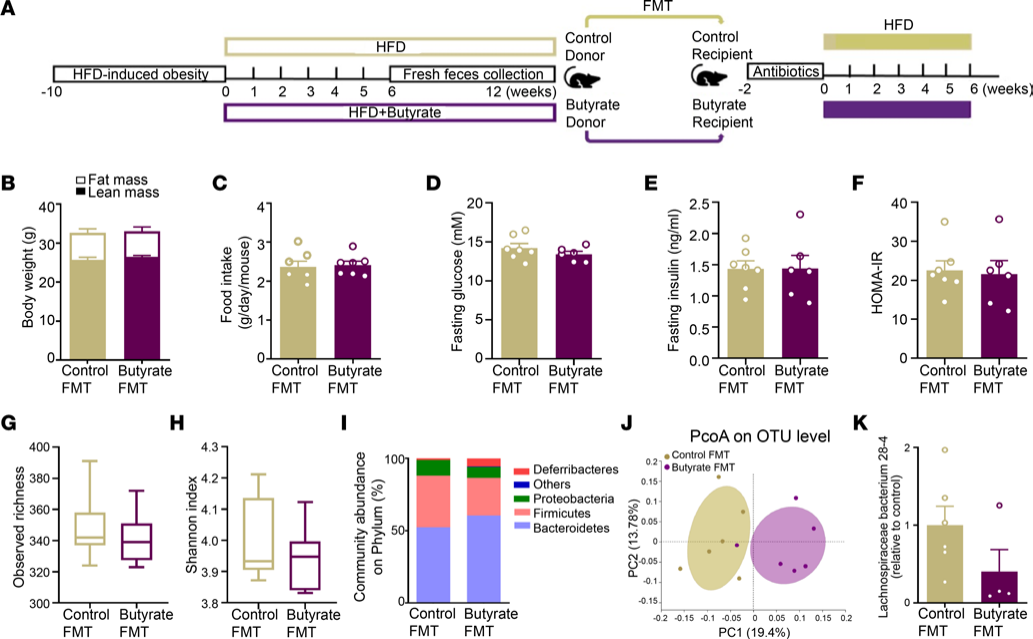
FMT from butyrate-treated obese donor mice does not attenuate weight gain, ameliorate metabolic health, or enrich *Lachnospiraceae bacterium 28-4* in recipient mice. DIO mice fed an HFD without or with 5% (w/w) butyrate treatment for 6 weeks. (**A**) After this, fresh feces were collected weekly and used for FMT to gut microbiota-depleted recipient mice that were fed an HFD for 6 weeks. (**B**) Body composition was calculated by MRI at the end of the study (*n* = 6–7). (**C**) The average food intake per day throughout the intervention period was calculated (*n* = 6–7). (**D**) Fasting plasma glucose (*n* = 6–7) and (**E**) insulin (*n* = 6–7) were measured and used to calculate (**F**) HOMA-IR (*n* = 6–7). Cecum bacterial DNA was collected and (**G**) the observed richness of taxonomy (*n* = 6) and (**H**) Shannon diversity (*n* = 6) of gut microbiota were calculated by 16S rRNA-Seq analysis. (**I**) The composition of abundant bacteria on phylum (*n* = 6) and (**J**) PCoA plot of unweighted UniFrac distances on OTU levels (*n* = 6) were calculated. (**K**) The abundance of *Lachnospiraceae bacterium 28-4* was quantified by real-time PCR (*n* = 4–6). Data are shown as means ± SEM for **B**–**F** and **K** or box plot with whiskers at min/max for **G** and **H**. Statistical significance between 2 groups was determined with 2-tailed Student’s unpaired *t* test; For data represented in the line graphs showing the changes over time for a continuous variable, statistical significance between 2 groups at each time point was determined using 2-tailed Student’s unpaired *t* test.

Finally, we assessed whether the inability of dietary butyrate to exert metabolic benefits in the treatment of DIO is related to the gut microbiota. To this end, DIO mice were first fed an HFD without or with butyrate treatment for 6 weeks. Then fresh fecal bacteria were isolated from obese donor mice that received the same HFD without or with butyrate between 6 and 12 weeks and transplanted to gut microbiota–depleted recipient mice (Figure 6A). In full support that butyrate does not ameliorate metabolic health in DIO mice (Figure 5, B–G), FMT from butyrate-treated obese donors did not affect BW and fat mass (Figure 6B), food intake (Figure 6C), or markers related to insulin resistance, including fasting plasma glucose, insulin, and HOMA-IR, in recipient mice compared with FMT from control donors (Figure 6, D–F). Although some differences in the composition rather than in the diversity of gut microbiota were observed in recipient mice from different groups (Figure 6, G–J), FMT from butyrate-treated obese donors did not affect the abundance of *Lachnospiraceae bacterium 28-4* in recipient mice (Figure 6K).

## Discussion

SCFA administration has been suggested as a potential therapeutic strategy to combat cardiometabolic diseases (14). In particular, dietary butyrate was previously demonstrated to reduce appetite (7), prevent DIO (5), and improve insulin sensitivity (6) in mice. However, the molecular mechanisms underlying these beneficial effects of dietary butyrate on cardiometabolic health are still unknown. Here, by performing a combination of AIMD and FMT, 2 widely used approaches to manipulate gut microbiota, we demonstrate that butyrate exerts its beneficial effects in a gut microbiota-dependent manner. In addition, our data hint toward the involvement of *Lachnospiraceae bacterium 28-4* in the metabolic benefits induced by dietary butyrate.

We previously revealed that dietary butyrate prevents the development of DIO by reducing appetite, which is related to a change in the gut microbiota composition (7). Thus, in the current study, we first investigated whether gut microbiota plays a causal role in these metabolic benefits of dietary butyrate. By performing AIMD, we demonstrated that eradication of bacterial gut microbiota (>95%) completely abolished the dietary butyrate-induced appetite reduction and BAT activation. In addition, we found that the predominant benefits of dietary butyrate on host energy metabolism, i.e., reducing appetite, ameliorating weight gain, and improving insulin resistance, were transferable by FMT, implying these metabolic benefits of dietary butyrate are causally dependent on the gut microbiota. While dietary butyrate activates BAT (7), in this study, FMT from butyrate-treated donors did not activate BAT in recipient mice, indicating that dietary butyrate probably activates BAT independent of gut microbiota, although this is in seeming opposition to the observation that AIMD abolished the BAT-activating effects of dietary butyrate. Gut microbiota depletion has been demonstrated to promote the browning of white adipose tissue and reduce obesity (15), indicating the depletion of gut microbiota per se might affect BAT activity. Indeed, we found that AIMD itself increases the uptake of triglyceride-derived (TG-derived) fatty acids by BAT and reduces the lipid content within brown adipocytes. Thus, based on the activated state of BAT resulting from AIMD, changes in gut microbiota induced by dietary butyrate were probably unable to promote BAT activation further.

Next, in search for the specific gut microbe change(s) that may contribute to the beneficial effects of dietary butyrate, we screened the gut microbiota of mice receiving FMT by metagenomic sequencing and discovered more than 800 significantly regulated species. Among the top 30 species based on relative abundance, the abundance of *Lachnospiraceae bacterium 28-4* was markedly increased by FMT from butyrate-treated donors. Of utmost interest is that the abundance of *Lachnospiraceae bacterium 28-4* negatively correlated with the host metabolic health parameters, including satiety, BW, and fasting glucose and insulin levels, indicating that the enrichment of *Lachnospiraceae bacterium 28-4* may be the key underlying the beneficial effects of dietary butyrate. *Lachnospiraceae bacterium 28-4* is a bacterium from an unclassified genus *Lachnospiraceae*; family Lachnospiraceae; order Clostridiales; class Clostridia; and phylum Firmicutes that was previously found in murine cecum content (16). The presence of *Lachnospiraceae bacterium 28-4* in the murine gut was recently confirmed by a single-cell resolution genome analysis (17). Furthermore, in a study of the evolution of mammals and their gut microbes, the genome of *Lachnospiraceae bacterium 28-4* was reported to be present in the fecal microbiota of Western lowland gorillas (18), indicating the possibility of the existence of this strain in the gut of primates. However, there is still limited information about the potential functions of this strain.

Predictive analysis of the microbiome using KEGG pathways indicated that *Lachnospiraceae bacterium 28-4* is enriched in enzymes involved in butyrate synthesis, as evidenced by the higher expression of genes coding butyrate kinase (EC2.7.2.7) and phosphate butyryltransferase (EC2.3.1.19) in recipient mice receiving FMT from butyrate-treated donors (Supplemental Figure 5, A–C). Accordingly, we cultured the cecal bacterial mixture from mice receiving butyrate-derived FMT containing a high abundance of *Lachnospiraceae bacterium 28-4* with ^13^C-labeled butyrate anaerobically for 7 days and tested whether exogenous butyrate could directly stimulate *Lachnospiraceae bacterium 28-4* to produce endogenous butyrate and other SCFAs. However, exogenous butyrate did not increase the production of endogenous SCFAs, including acetate, propionate, and butyrate (Supplemental Figure 5, E and F). In line with this, FMT from butyrate-treated donors did not increase the concentration of cecal butyrate in recipient mice (Supplemental Figure 5D) either. Although previous studies indicated that butyrate is capable of being converted to acetate by gut microbiota (19), we did not observe that adding butyrate into *Lachnospiraceae bacterium 28-4* could increase ^13^C-acetate levels (Supplemental Figure 5F). Collectively, these in vitro and in vivo findings indicate that *Lachnospiraceae bacterium 28-4* per se do not produce butyrate and other SCFAs in measurable quantities. As an alternative approach to identify the mechanism by which FMT from butyrate-treated mice is linked to the beneficial metabolic outcome, we performed untargeted metabolomics on the cecal content samples of recipient mice receiving FMT and found that the abundance of *Lachnospiraceae bacterium 28-4* was markedly associated with several metabolites in the gut. Further investigation on whether cultured purified *Lachnospiraceae bacterium 28-4* could produce those metabolites, which may serve as the dominant mediator contributing to its metabolic benefits, would be of interest.

In the current study, we observed that dietary butyrate did not restore metabolic health in DIO mice, which is in full agreement with the previous findings that oral butyrate only improves insulin sensitivity in individuals who are healthy rather than metabolically compromised (8). The differences in response to dietary butyrate of the lean versus obese mice may be explained by the insufficient ability of dietary butyrate to improve obesity-induced dysbiosis. In contrast to the increased abundance of *Lachnospiraceae bacterium 28-4* in the prevention strategy, dietary butyrate failed to modulate the abundance of this bacteria in the treatment of DIO mice (Supplemental Figure 5, G–J). Furthermore, FMT from butyrate-treated obese donors containing a very low abundance of *Lachnospiraceae bacterium 28-4* did not improve metabolic health in recipient mice. In line with this, a recent study showed that fibers affect metabolic health in lean but not in prediabetic men, which may be associated with specific changes in the gut microbiota (20). It has been shown that orally administered sodium butyrate can be efficiently absorbed in the small intestine (21), which may establish a favorable environment in the small intestine for *Lachnospiraceae bacterium 28-4* growth. Indeed, butyrate has been shown to enhance proliferation, differentiation, and maturation, and rescue apoptosis of enterocytes in the small intestine, thereby influencing the small intestinal environment (22). In contrast, consumption of HFD induces the production of ROS (23), an essential pathogenic factor in the gut (24) that triggers HFD-induced oxidative stress and gut microbiota dysbiosis (25). Of note, SCFAs inhibit ROS production and protect against oxidative stress (26). Taken together, we suggest that in lean mice, HFD-induced ROS and oxidative stress can be restored by butyrate administration and improvement of gut microbiota. However, in obese mice, long-term HFD induces severe oxidative stress that cannot be reversed by butyrate and prevents the outgrowth of *Lachnospiraceae bacterium 28-4* in the gut. Thus, the abundance of *Lachnospiraceae bacterium 28-4* may precisely predict whether dietary butyrate can beneficially affect cardiometabolic health. In addition, strategies to increase the richness of *Lachnospiraceae bacterium 28-4* in the gut may provide personalized therapeutic handles to combat obesity and associated cardiometabolic diseases in specific subgroups and, ultimately, in the individual.

A proof-of-concept study demonstrated the feasibility of the administration of a specific bacterial strain, *Akkermansia muciniphila*, to improve insulin resistance in humans, providing a promising start for developing future clinical interventions with gut microbiota (27). However, we still have some obvious hurdles to overcome to assess the application of *Lachnospiraceae bacterium 28-4* as a potentially novel probiotic in reducing appetite and combating obesity and associated metabolic diseases. In fact, despite avid attempts to amplify *Lachnospiraceae bacterium 28-4* in vitro under anaerobic conditions from material obtained during our animal experiments, we were unable to isolate a pure culture of *Lachnospiraceae bacterium 28-4*, as *Lachnospiraceae bacterium 28-4* is very sensitive to oxygen. In addition, it is also probably due to the overgrowing of other bacteria that slows the growth of *Lachnospiraceae bacterium 28-4*.

In summary, we revealed a crucial role of gut microbiota in the beneficial metabolic effects of dietary butyrate, including decreased food intake, prevented DIO and improved insulin resistance, all of which are strongly associated with the abundance of *Lachnospiraceae bacterium 28-4* in the gut. Future research should focus on optimizing culturomics strategies for obtaining pure *Lachnospiraceae bacterium 28-4* to prove their causal role in the metabolic health effects of dietary butyrate. Given members of the Lachnospiraceae family, such as *Blautia* (28) and *Roseburia* (29, 30), have been negatively associated with obesity and metabolic disorders in the clinic, our findings provide leads for next-generation probiotic development, providing a potential therapeutic strategy to combat obesity and its associated metabolic diseases in humans. This is particularly relevant, as a very recent study showed that oral butyrate supplementation to children with obesity decreased BMI, waist circumference, insulin, and HOMA-IR (31).

## Methods

### Animals.

Male E3L.CETP mice expressing the human APOE*3-Leiden and CETP genes were generated as previously described (32). Mice that were 12–16 weeks old were used and housed under standard conditions with a 12 hour light/dark cycle (0700–1900) and with ad libitum access to regular chow and water unless indicated otherwise. For each experiment, mice were randomly divided into groups based on their BW and plasma parameters (total cholesterol, TGs, glucose, and free fatty acids). Randomization of location was used to minimize potential confounders. No criteria were set for excluding animals during the experiments.

### Gut microbiota depletion.

For AIMD of the gut, lean mice initially received a 200 μL antibiotic cocktail (0.5 mg/mL ampicillin, 0.5 mg/mL neomycin, and 0.5 mg/mL metronidazole; Sigma-Aldrich; and 0.25 mg/mL vancomycin; Xellia Pharmaceuticals) by oral gavage once a day for 1 week, which was followed by administration of these antibiotics in their drinking water (0.25 mg/mL ampicillin, 0.25 mg/mL neomycin, 0.25 mg/mL metronidazole, and 0.125 mg/mL vancomycin) for the next 5 weeks. The control group received saline by oral gavage for 1 week followed by regular drinking water for 5 weeks. At the same time, mice were fed with an HFD (60% high fat and 0.25% cholesterol; Altromin) without or with 5% (w/w) sodium butyrate (Sigma-Aldrich).

### DIO mice.

To induce obesity in mice for subsequent experiments, lean mice were fed an HFD containing 60% high fat and 0.25% cholesterol (Altromin) for 10 weeks.

### Dietary butyrate prevention and treatment study.

To assess the prevention and treatment effects of dietary butyrate on DIO, lean and DIO mice received an HFD without or with 5% (w/w) sodium butyrate (i.e., on average, 0.12 g per day per mouse) for 6 weeks. This 5% w/w sodium butyrate diet has been previously published (6, 7). Since addition of sodium butyrate to the diet might induce the diet energy dilution, and butyrate is also a kind of energy source, we have measured the total energy content of the HFD without or with butyrate using bomb calorimetry. We found the addition of sodium butyrate to the diet somewhat decreased the energy content by 7%, compared with that of the control diet. The addition of 5% w/w sodium butyrate has been reported to decrease food intake by as much as 22% (7), indicating that the energy dilution induced by adding sodium butyrate to the feed has a minor contribution to metabolic benefits induced by butyrate.

### Conditioned taste aversion experiment.

The experimental setup was designed according to a modified procedure as described previously (33). In brief, mice were housed under standard conditions with ad libitum access to regular chow diet and water. In the first week, mice received saline or butyrate by oral gavage every other day. After the oral gavage, a drinking bottle filled with sweeteners containing either 1 M L-serine (saline group) or 0.6 M sucrose (butyrate group) were put on the cages for 1 hour, respectively. During this period, mice had free access to 2 drinking bottles. On the first day of the second week, mice were housed under standard conditions with ad libitum access to chow diet and water containing L-serine or sucrose. The consumption of water solution of L-serine and sucrose was calculated after 24 hours (for detailed procedures, Supplemental Figure 6A). We clearly showed that oral butyrate does not influence the preference of butyrate-treated mice for sucrose solution (Supplemental Figure 6B), indicating that butyrate does not affect the feeding behavior of mice.

### FMT.

Both lean (prevention) and DIO (treatment) mice received an HFD without or with 5% (w/w) sodium butyrate for 6 weeks. After this, when a stable gut microbiota community had developed (34), fresh feces were collected weekly for another 6 weeks for FMT. Pellets of those feces were diluted in Ringer’s solution supplemented with 0.05% l-cysteine hydrochloride (both Sigma-Aldrich). The fecal supernatant was centrifuged and filtered through a 100 μm cell strainer (Corning) for subsequent FMT. In recipient mice, AIMD was first performed as detailed above, albeit 1 week by oral gavage followed by 1 week via drinking water. Subsequently, mice were subjected to FMT by oral gavage with 200 μL microbiota from a specific donor group 3 times a week for 6 weeks. The residual fecal supernatant was stored at –80°C until further analysis.

### BW and body composition.

BW was measured with a scale. Body composition was measured in conscious mice using an EchoMRI-100.

### Plasma parameters.

At the end of each experiment, venous blood samples of approximately 100 μL were collected from the tails of 5-hour fasting mice (0800–1300) using heparin-coated capillary tubes. Plasma was isolated after centrifugation (12,000 rpm, 5 minutes, at room temperature) and stored at –80°C. Plasma glucose was measured using a commercially available enzymatic kit (Roche Diagnostics), and plasma insulin was determined with an ultrasensitive mouse insulin ELISA kit (Crystal Chem). HOMA-IR was calculated with the equation for mice: IR_HOMA_ = [insulin] (unit)/(22.5 × e^-In[(glucose)^ ^[unit]]^) as described (35).

### Indirect calorimetry.

Energy expenditure of individually housed mice was measured by indirect calorimetry using automatic metabolic cages (Sable Systems) for 5 consecutive days. After 2 days of acclimatization, oxygen consumption and carbon dioxide production were recorded. The average respiratory exchange ratio, fat oxidation rate, and carbohydrate oxidation rate were calculated from day 3 to day 5, as described (36).

### In vivo lipid clearance.

When indicated, mice were intravenously injected via the tail vein with 80 nm glycerol [^3^H]TO-labeled TRL-like particles (1 mg TG in 200 μL) (37). Blood samples were taken from the tail vein at 2, 5, 10, and 15 minutes to assess TG clearance by counting of ^3^H activity in 10 μL plasma. Subsequently, mice were sacrificed by CO_2_ inhalation and were perfused via the heart with ice-cold saline for 5 minutes. Pieces of the collected organs (approximately 50 mg) were weighed and dissolved in tissue Solubilizer (Amersham Biosciences) overnight at 56°C. ^3^H activity in both plasma and organ samples was determined using scintillation counting (TRI-CARB, PerkinElmer) after addition of liquid scintillation cocktail (Ultima Gold, PerkinElmer).

### In vitro cecal bacteria culture.

Cecal bacteria were isolated from mice and cultured anaerobically at 37°C in the yeast extract, casitone, and fatty acid (YCFA) culture medium without or with 4 mM butyrate sodium for 3 or 7 days. At the end of the culture, bacterial DNA was isolated and the abundance of *Lachnospiraceae bacterium 28-4* was assessed by real-time PCR.

For the endogenous SCFA production study, cecal bacteria were isolated from mice and cultured in the YCFA culture medium without or with additional ^13^C-labeled butyrate (0, 4, 20, and 50 mM) supplementation anaerobically for 7 days. At the end of the experiment, the culture medium was collected for quantification of ^12^C- and ^13^C-SCFAs.

### SCFA measurement.

The SCFAs in the cecal samples from mice were measured by gas chromatography/mass spectrometry (GC/MS) using previously published approach with modifications (38). Briefly, 10 μL of plasma was transferred to a glass vial containing 250 μL acetone (Sigma-Aldrich), 1 mg/L internal standards solution containing acetic acid-d4, propionic acid-6, and butyric acid-d8 (Sigma-Aldrich), as well as 10 μL of ethanol. Thereafter, samples were derivatized with pentafluorobenzyl bromide (PFBBr), as follows: 100 μL 172 mM PFBBr (Thermo Fisher Scientific) in acetone was added, samples were mixed, and heated to 60°C for 30 minutes. After the samples had cooled down to room temperature, a liquid-liquid extraction was performed using 500 μL n-hexane (Sigma-Aldrich) and 250 μL GC/MS-grade water. The upper n-hexane layer was transferred to a fresh glass vial and subsequently used for GC/MS analysis. Analogous calibration standards were prepared. For calibration standards, no plasma was added and 10 μL of ethanol (EtOH) was replaced by 10 μL standards solution (Sigma-Aldrich) in EtOH. Samples were analyzed on a Bruker Scion 436 GC fitted with an Agilent VF-5ms capillary column (25 m × 0.25 mm inner diameter, 0.25 μm film thickness) coupled to a Bruker Scion TQ MS. Injection was performed using a CTC PAL autosampler (G6501-CTC): a 1 μL sample was injected splitless at 280°C. Helium 99.9990% was used as carrier gas at a constant flow of 1.20 mL/min. The GC temperature program was set as follows: 1 minute constant at 50°C, then linear increase at 40°C/min to 60°C, kept constant for 3 minutes, followed by a linear increase at 25°C/min to 200°C, linearly increased at 40°C/min to 315°C, and then kept constant for 2 minutes. The transfer line and ionization source temperature were 280°C. The pressure of the chemical ionization gas, methane (99.9990%), was set at 15 psi. Negatively charged ions were detected in the selected ion monitoring mode, and acetic acid, acetic acid-d4, propionic acid, propionic acid-d6, butyric acid, and butyric acid-d8 were monitored at *m/z* 59, 62, 73, 78, 87, and 94, respectively.

The ^12^C- and ^13^C-labeled SCFAs from culture medium were measured by NMR. An aliquot of 225 μL sample was mixed with 25 μL of 1.5 M K_2_HPO_4_/KH_2_PO_4_ (Sigma-Aldrich) solution (pH = 7.4) in 99.9% deuterated water (D_2_O; CortecNet) containing 0.2 mM NaN_3_ (Sigma-Aldrich) and 0.05 mM of trimethylsilylpropionic-d4 acid sodium salt (TSP-*d*_4_; Sigma-Aldrich). Next, samples were analyzed by NMR spectroscopy using a 14.1T NMR spectrometer (600 MHz for ^1^H; Bruker Avance Neo) under standardized instrumental settings for all samples (39). A 1-dimensional (1D) ^1^H spectrum was collected using the *noesygppr1d* pulse sequence. All spectra data were phased, baseline corrected, and referenced to TSP-*d*_4_ methyl protons at δ 0.00 ppm and subsequently imported into Chenomx NMR suit 9.0 for the quantification of SCFAs. ^13^C-acetate was quantified using the ^1^H-^13^C Heteronuclear Single Quantum Correlation experiment and calculated by background corrections.

### BAT histology.

Interscapular BAT was isolated, fixed with formalin, and embedded in paraffin. BAT sections (5 μm), prepared using a microtome (Leica RM2245), were stained with H&E and IHC stained for UCP-1 (1/4,000; catalog ab10983, Abcam) and TH (1/2,000; catalog ab134461, Abcam) as previously described (40). Quantification of the intracellular lipid area, UCP-1 protein, and TH protein within BAT was performed using ImageJ software (Version 1.50i; NIH). Results were expressed as percentage of positive area versus total BAT area.

### Genomic DNA extraction.

At the end of the experiments, cecum contents were collected in sterile Eppendorf tubes. Genomic bacterial DNA was isolated from cecum samples with fast DNA stool mini kits (QIAamp, QIAGEN) following the manufacturer’s instructions.

### 16S rRNA-Seq processing and data analysis.

Once we collected the DNA samples, a quality test was first performed. Then, all the qualified DNA was used to construct libraries. We used fusion primer with dual index and adapters for PCR. Fragments too short would be removed by Ampure beads. Only the qualified library can be used for sequencing. The paired-end reads were generated using the Illumina MiSeq (BGI Genomics). Briefly, the reads with, e.g., sequencing adapters, N base, poly base, and low quality, were filtered out with default parameters to generate clean reads. Overlapping paired-end reads were generated by Fast Length Adjustment of Short reads (FLASH, v1.2.11) (41) and merged to tags. Next, the tags were clustered to OTU by scripts of software USEARCH (v7.0.1090) at 97% sequence similarity (42). OTU representative sequences were taxonomically classified using Ribosomal Database Project (RDP) Classifier v.2.2 trained on the SILVA database (Release 128), using 0.7 confidence values as the cutoff. We analyzed α- and β-diversity based on OTU using the free online Majorbio I-Sanger Cloud Platform (https://cloud.majorbio.com). In brief, α-diversity metrics of observed richness were calculated using mothur (v1.30.1) and visualized with GraphPad Prism (v8.0, box plot, whiskers at min/max). Several β-diversity metrics were determined by QIIME and visualized with Prism GraphPad. PCoA based on Bray-Curtis was visualized using R. Significance of clustering was determined by Bray-Curtis distance matrices.

### Metagenomic sequencing and processing.

Once we collected the DNA samples, a quality test was first performed. Qualified bacterial DNA samples were first sheared into smaller fragments by nebulization. Then the overhangs resulting from fragmentation were converted into blunt ends using T4 DNA polymerase, Klenow Fragment, and T4 Polynucleotide Kinase. After adding an adenine (A) base to the 3’ end of the blunt phosphorylated DNA fragments, adapters were ligated to the ends of the DNA fragments. Then, short fragments were removed with Ampure beads. Agilent 2100 Bioanalyzer and ABI StepOnePlus Real-Time PCR System were used to qualify and quantify the sample libraries. Finally, the qualified libraries were sequenced using Illumina HiSeqTM4000 according to the workflow specified by the service provider (BGI Genomics). The raw sequencing data were deposited in the NCBI’s Sequence Read Archive (SRA reference 12525537). Reads smaller than 50 bp and gene contamination were further removed by mapping the reads against the host reference genome through the software Burrows-Wheeler Aligner (43) and removed and compared with highly similar polluted reads. On average, 4.8 Gbp clean data per sample were generated. Clean reads were then de novo assembled using Multiple–MEGAHIT (44). MetaGeneMark (v2.10) (45) was then used to predict the bacterial ORFs from assembled contigs of at least 500 bp. CD-HIT software (v4.5.8) was used to exclude the redundant genes from all predicted ORFs to construct a preliminary nonredundant gene catalog (46). The predicted ORFs with lengths over 100 bp were translated into aa sequences via NCBI ORF finder, which were subsequently blasted against public databases including NCBI-NR and KEGG (47) to obtain taxonomic and metabolic functional annotation.

### Metagenomics data analysis.

Based on the profile abundance, analysis of differences in microbial taxonomy and functional enrichment were then performed using free online Majorbio I-Sanger Cloud Platform. Briefly, statistical significance on species level was determined with unpaired 2-tailed Student’s *t* test or LEfSe with standard parameters (linear discriminant analysis score 2.0). Relationships between abundance of gut microbe (top 30 based on relative abundance) and metabolic parameters were presented using Spearman’s correlation heatmap in R package of heatmap.

RDA demonstrating the relationships between gut microbe distribution (top 10 based on relative abundance) and metabolic parameters was visualized using R with vegan package. Statistical significance on bacterial functional contribution mapping with KEGG level 2 was determined using unpaired 2-tailed Student’s *t* test and visualized with heatmap using R and Python with package of NetworkX. The butyrate kinase (2.7.2.7) and phosphate butyryltransferase (2.3.1.19) mapping with KEGG pathway (butanoate metabolism, map00650) were presented as a partial pathway adapted from the KEGG database, the statistical significance of which was determined using unpaired 2-tailed Student’s *t* test.

### Untargeted metabolomics sequencing and data analysis.

The untargeted metabolomics were performed using liquid chromatography-tandem mass spectrometry (LC-MS/MS) technology with high-resolution mass spectrometer Q Exactive (Thermo Fisher Scientific) to collect data from both positive and negative ions to improve metabolite coverage. LC-MS/MS data processing was performed using the Compound Discoverer 3.1 (Thermo Fisher Scientific) software, which mainly included peak extraction, peak alignment, and compound identification. Data preprocessing, statistical analysis, metabolite classification annotations, and functional annotations were performed using the self-developed metabolomics R package metaX (48) and the metabolome bioinformatic analysis pipeline by BGI. The multivariate raw data are dimensionally reduced by PCoA to analyze the groupings, trends (intra- and intergroup similarities and differences) and outliers of the observed variables in the data set (whether there is an abnormal sample). Using partial least squares method-discriminant analysis (PLS-DA), the VIP values of the first 2 principal components of the model, combined with the variability analysis, the fold change, and the 2-tailed Student’s *t* test, were used to screen for differential metabolites. Differential metabolites screening conditions include 1) VIP of the first 2 principal components of the PLS-DA model greater than or equal to 1; 2) fold change greater than or equal to 1.2 or less than or equal to 0.83; and 3) *P* value less than 0.05. Metabolic pathway enrichment analysis of differential metabolites was performed based on the KEGG database. Metabolic pathways with a *P* value less than 0.05 were significantly enriched by differential metabolites.

### 16S rRNA and Lachnospiraceae bacterium 28-4 real-time PCR.

PCR amplification targeting the V4 region of bacterial 16S rRNA was performed using the highly efficient and universal primers (926F) 5′ AAACTCAAAKGAATTGACGG 3′ and (1062R) 5′ CTCACRRCACGAGCTGAC 3′. The primers above were also used as control of the real-time PCR amplification identifying *Lachnospiraceae bacterium 28-4* using the house-designed primers 5′ GGGTGTACAGAAGGGAAGATTACG 3′ and 5′ AAACTCCGGTGGTACAGGATG 3′. For the validation of the primers of *Lachnospiraceae bacterium 28-4*, PCR products were purified and cloned using the pGEM-T and pGEM-T Easy Vectors Systems (detailed procedures are in Technical Manual TM042, Promega). Next, the selected colonies were grown in culture medium overnight in a 37°C incubator roller drum for 12 hours, and subsequently the DNA of the colonies was prepared and sequenced. Finally, we blasted the sequence using Nr/Nt/16s SILVA database and indeed found the sequence belonging to the *Lachnospiraceae bacterium 28-4* (query cover 100%, identity 99.86%).

### Statistics.

Data are expressed as mean ± SEM or box plot, whiskers at min/max, unless indicated otherwise. Statistical significance between 2 groups was determined with 2-tailed Student’s unpaired *t* test or Wilcoxon’s rank-sum test unless indicated otherwise. For data represented in the line graphs showing the changes over time for a continuous variable, statistical significance between 2 groups at each time point was determined using 2-tailed Student’s unpaired *t* test. All statistical analyses were performed using Prism 8 (GraphPad Software). *P* < 0.05 was considered to be significant.

### Study approval.

All animal experiments were performed under approval by the Ethics Committee on Animal Care and Experimentation of the Leiden University Medical Center and following the regulations of Dutch law on animal welfare.

## Author contributions

ZL conceptualized and designed the study; contributed to the acquisition of data; analyzed and interpreted the data; and drafted, edited, and revised the manuscript. EZ, CL, HW, SY, FR, ZY, SK, DPYK, MH, SK, MG, IMJGS, EJK, WKS, and KWVD contributed to the acquisition of data; analyzed and interpreted the data; and edited and revised the manuscript. PCNR and YW conceptualized and designed the study; obtained funding; supervised the study; and edited and revised the manuscript.

## Supplementary Material

Supplemental data

Supplemental table 1

Supplemental table 2

Supplemental table 3

Supplemental table 4

## Version 1 02/22/2023

Electronic publication
