# Supplementary material for: Dietary butyrate ameliorates metabolic health associated with selective proliferation of gut Lachnospiraceae bacterium 28-4
Source: JCI Insight. 2023 Feb 22;8(4):e166655. doi: 10.1172/jci.insight.166655 (PMC9977501; doi:10.1172/jci.insight.166655)
Supplement: Supplemental data [file jciinsight-8-166655-s119.pdf]

Supplemental Information

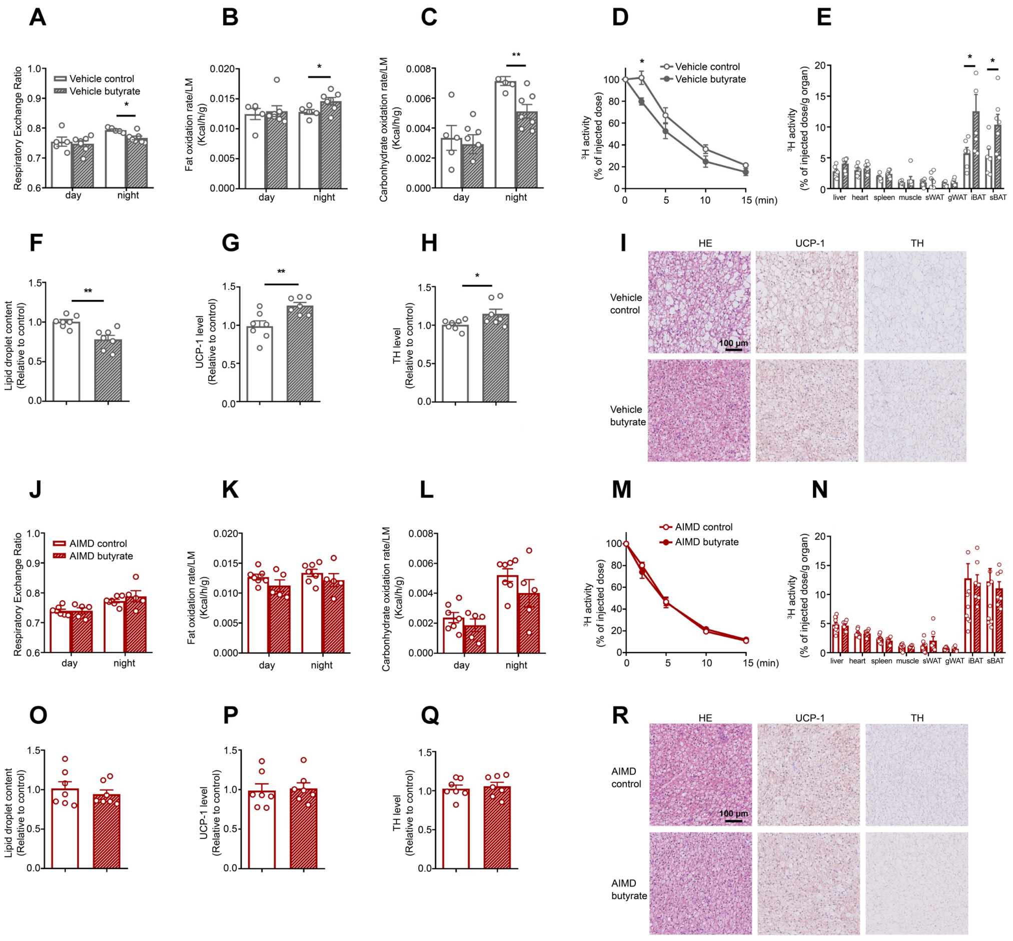

Figure S1. Dietary butyrate increases fat oxidation accompanied by parameters of BAT activation dependent on gut microbiota.

Mice received antibiotics-induced microbiota depletion (AIMD) or saline (Vehicle) for six weeks while being fed a high-fat diet (HFD) without or with 5% (w/w) sodium butyrate. In the second week, mice were individually housed in automatic metabolic cages to assess energy expenditure by indirect calorimetry. Respiratory exchange ratio (A, J, n=5-7), fat oxidation (B, K, n=5-7) and carbohydrate oxidation (C, L, n=5-7) were calculated from data obtained during 3 consecutive days. Just before termination, mice were intravenously injected with glycerol tri[^{3}H]oleate-labeled triglyceride-rich lipoprotein-like particles, and ^{3}H-activity was assessed in plasma (D, M, n=6) and various organs (E, N, n=6). Interscapular brown adipose tissue (iBAT) was isolated and used for immunohistochemistry staining (F-I, O-R, n=7). Lipid content (F, O), uncoupling protein-1 (UCP-1) protein content (G, P) and tyrosine hydroxylase (TH) protein content (H, Q) were quantified as representative pictures shown (I, R). Data are shown as means ± SEM; Statistical significance between two groups was determined with two-tailed Student unpaired t-test; For data represented in the line graphs showing the changes over time for a continuous variable, statistical significance between two groups at each time point was determined using two-tailed Student unpaired t-test; *P<0.05, **P<0.01; Butyrate vs Control. gWAT, gonadal white adipose tissue; HE, hematoxylin and eosin; LM, lean body mass; sBAT, subscapular brown adipose tissue; sWAT, subcutaneous white adipose tissue.

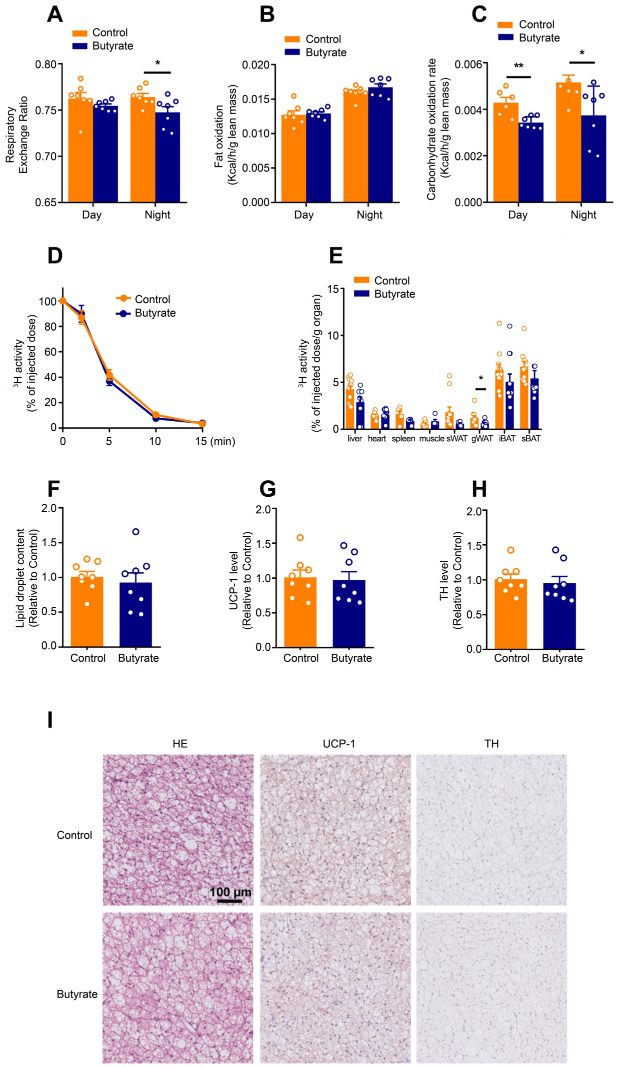

Figure S2. Fecal microbiota transplantation from butyrate-treated lean donor mice does not affect fat oxidation and brown adipose tissue activation in recipient mice.

Mice were fed a high-fat diet (HFD) without or with 5% (w/w) sodium butyrate prevention for 6 weeks. After this, fresh feces were collected weekly, and used for fecal microbiota transplantation (FMT) to gut microbiota-depleted recipient mice that were fed a HFD for 6 weeks. In the second week, mice were individually housed in automatic metabolic cages for 3 consecutive days to assess energy expenditure by indirect calorimetry measurement, and respiratory exchange ratio (A, n=7), fat oxidation (B, n=7) and carbohydrate oxidation (C, n=7) were calculated. Just before termination, mice we were intravenously injected with glycerol tri[^{3}H]oleate-labeled triglyceride-rich lipoprotein-like particles, and ^{3}H-activity was assessed in plasma (D, n=8) and various organs (E, n=8). iBAT was collected and used for immunohistochemistry staining, and lipid content (F, n=8), UCP-1 protein (G, n=8) and TH protein (H, n=8) was quantified as presentative pictures shown (I). Data are shown as means ± SEM; Statistical significance between two groups was determined with two-tailed Student unpaired t-test; For data represented in the line graphs showing the changes over time for a continuous variable, statistical significance between two groups at each time point was determined using two-tailed Student unpaired t-test. *P<0.05, **P<0.01; Butyrate vs Control.

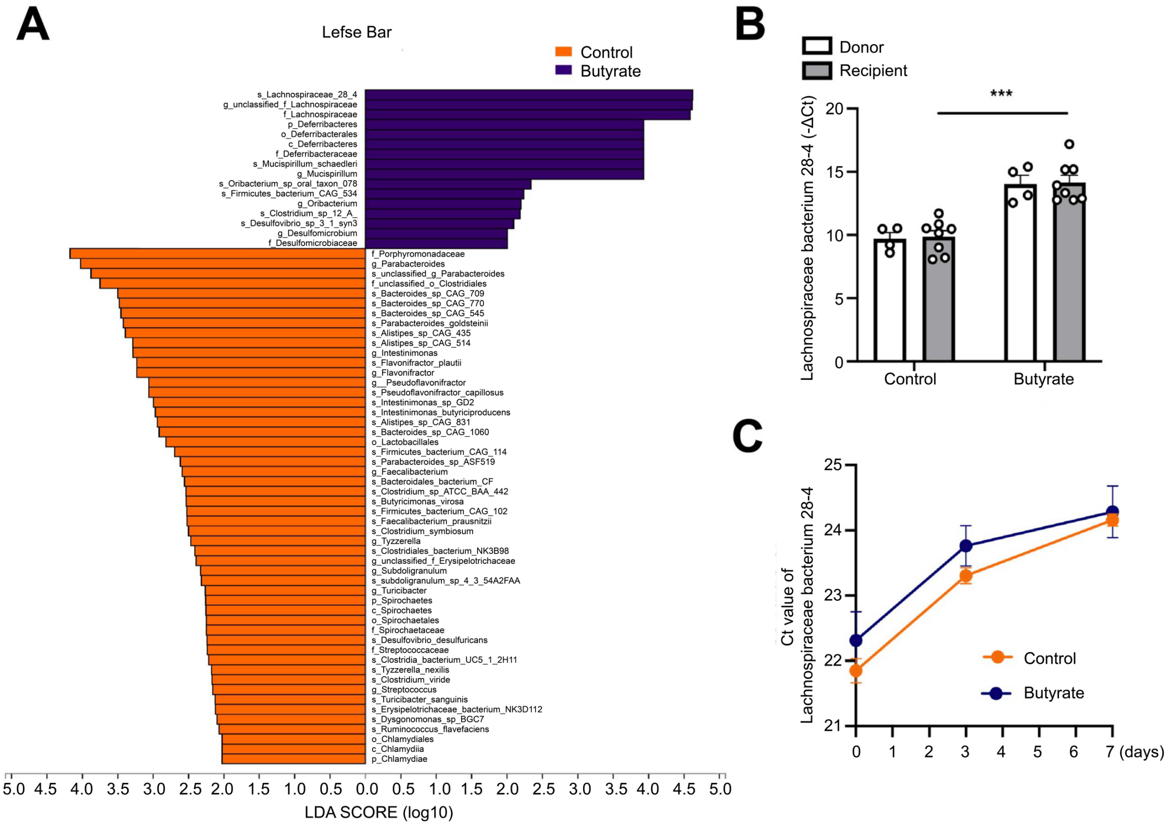

Figure S3. Fecal microbiota transplantation from butyrate-treated lean donor mice selectively enriches *Lachnospiraceae bacterium 28-4* in recipient mice.

Linear discriminant analysis (LDA) score of taxonomic cladogram was obtained from linear discriminant analysis effect size (LEfSe) analysis of metagenomics sequencing data (A, n=5). Gene of *Lachnospiraceae bacterium 28-4* was quantified by real-time PCR from cecal bacteria samples of donor mice (n=4) and recipient ones (B, n=8) and bacterial samples cultured *in vitro* (C, n=5). Data are shown as means ± SEM (B and C); Statistical significance between two groups was determined with two-tailed Student unpaired t-test; For data represented in the line graphs showing the changes over time for a continuous variable, statistical significance between two groups at each time point was determined using two-tailed Student unpaired t-test; ***P<0.001; Butyrate vs Control.

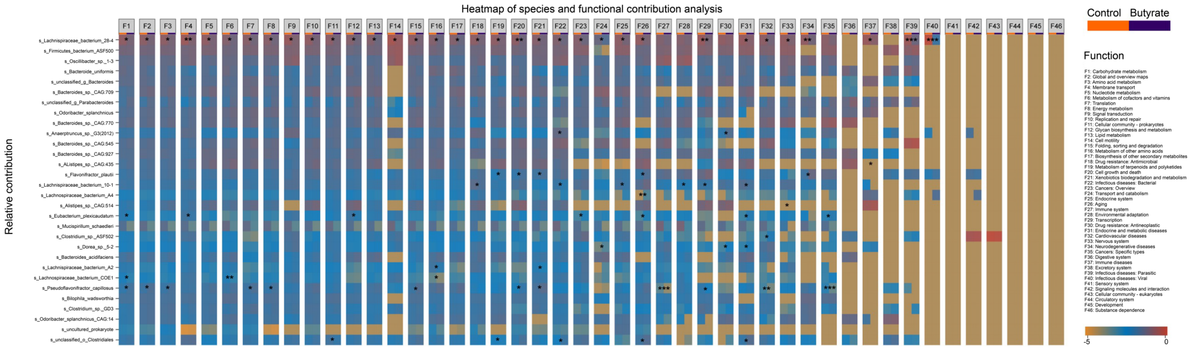

Figure S4. Gut microbiota functional contribution analysis.

Functional contributions of the gut microbiota (top 30 based on relative abundance) were analyzed using the Kyoto Encyclopedia of Genes and Genomes (KEGG) database (Pathway level 2). Statistical significance between two groups was determined with Wilcoxon rank-sum test; *P<0.05, **P<0.01, ***P<0.001; Butyrate vs Control.

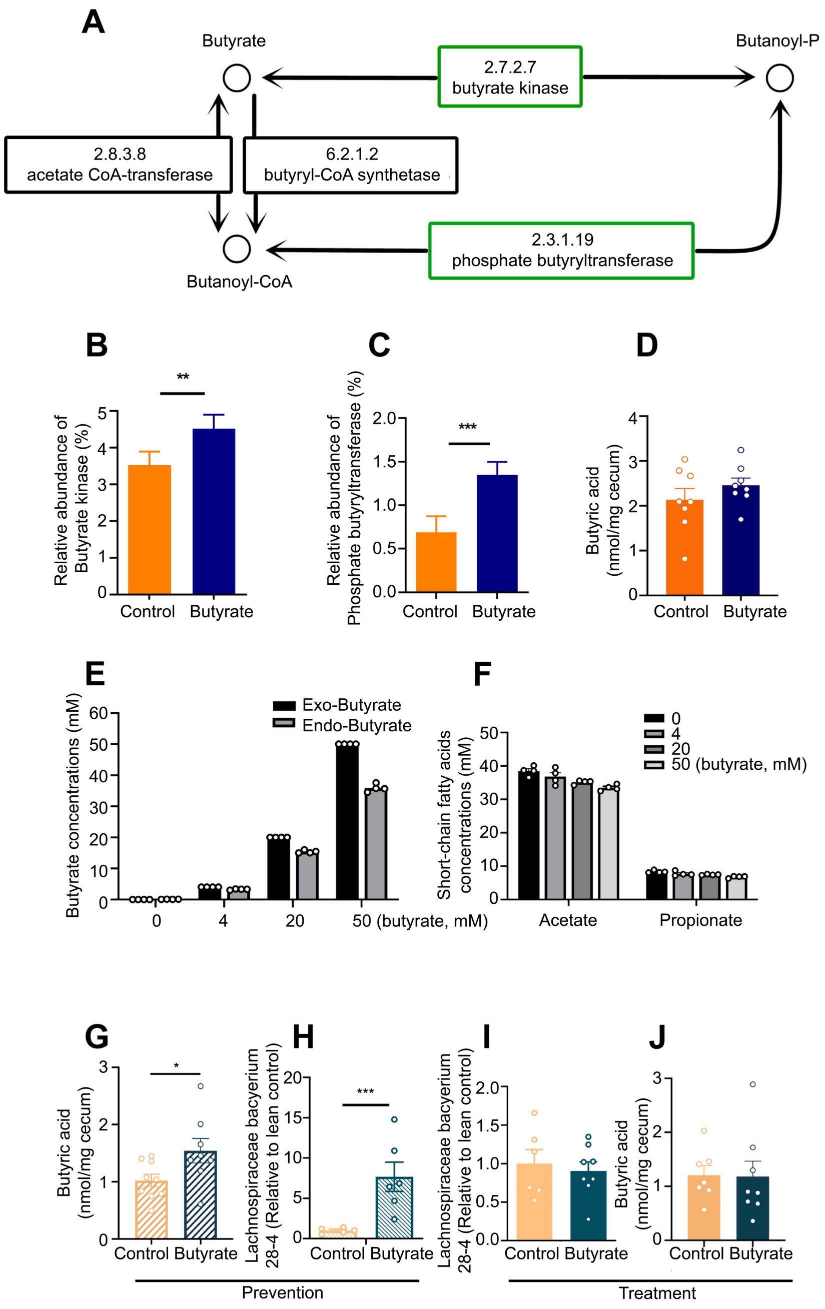

Figure S5. Dietary butyrate does not increase the endogenous butyrate production related to increased *Lachnospiraceae bacterium 28-4*.

Partial pathway of butyrate (butanoate) metabolism with a green box to highlight genes was adapted from Kyoto Encyclopedia of Genes and Genomes (KEGG) pathway database (A). The expression of genes coding butyrate kinase (B, n=5) and phosphate butyryltransferase (C, n=5) were quantified using KEGG pathway database. The concentrations of SCFAs within the cecum samples of mice (D, G and J, n=7-8) and *in vitro* culture medium (E and F, n=4) were measured by NMR. The gene of *Lachnospiraceae bacterium 28-4* within the cecum samples of mice receiving butyrate prevention (H) or treatment (I) was quantified by real-time PCR (n=5-8). Data are shown as means ± SEM; Statistical significance between two groups was determined with two-tailed Student unpaired t-test. *P<0.05, **P<0.01, ***P<0.001; Butyrate vs Control.

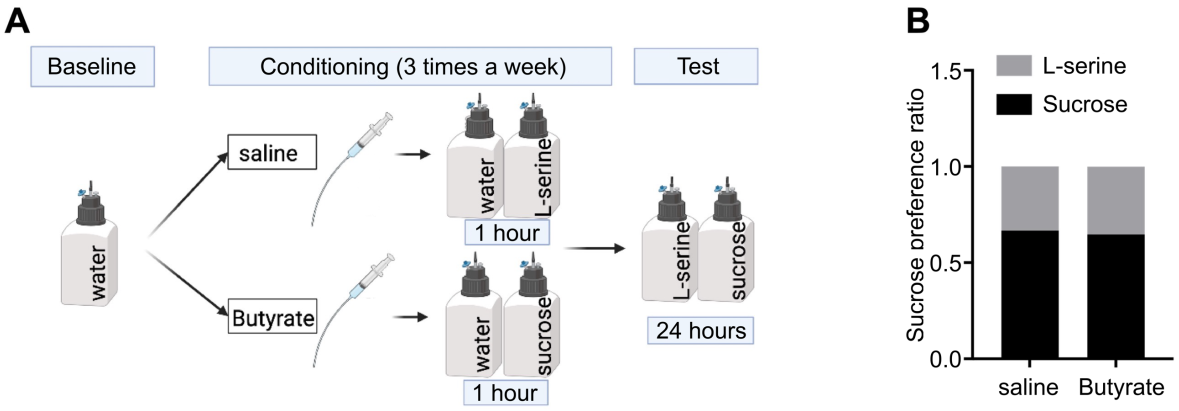

Figure S6. Dietary butyrate does not affect the feeding behavior of mice.

Detailed procedures of conditioned taste aversion experiment were presented (A). The proportion of sucrose consumption was calculated (B, n=5).
